# Serial assessment of inflammatory parameters for prediction of septic complications following surgery for colorectal endometriosis: A descriptive, retrospective study

**DOI:** 10.1007/s00508-021-01916-w

**Published:** 2021-08-02

**Authors:** Eliana Montanari, Lena Maria Reh, Bernhard Dauser, Tudor Birsan, Gernot Hudelist

**Affiliations:** 1 Department of Gynecology, Center for Endometriosis, Hospital St. John of God Vienna, Vienna, Austria; 2 Department of Obstetrics and Gynecology, Medical University of Vienna, Vienna, Austria; 3 Department of General Surgery, Center for Endometriosis, Hospital St. John of God, Vienna, Austria; 4 Stiftung Endometrioseforschung (SEF), Westerstede, Germany

**Keywords:** Anastomotic leakage, Body temperature, C‑reactive protein, Deep endometriosis bowel surgery, White blood cell count

## Abstract

**Purpose:**

To assess whether C‑reactive protein (CRP), white blood cell count (WBC) and body temperature changes are suitable parameters for the early detection of septic complications following resection of colorectal deep endometriosis (DE).

**Methods:**

Retrospective data analysis of CRP, WBC and body temperature courses following colorectal surgery for DE at a tertiary referral center for endometriosis.

**Results:**

Out of 183 surgeries performed, 10 major surgical complications were observed, including 4 anastomotic leakages (AL 2%) and 2 rectovaginal fistulae (RVF 1%). In the presence of a lower gastrointestinal tract (GIT)-related septic complication or abdominal wall abscess, serum CRP levels were increased starting at postoperative day 2–3. A cut-off value of 10 mg/dl on day 4 for prediction of early septic complications could be verified (area under the curve 0.94, obtained by receiver operating characteristics analysis, sensitivity 88%, specificity 90%, positive predictive value 32%, negative predictive value 99%). Additionally, most patients with early septic complications exhibited increased WBC levels starting mainly from day 3–4; however, increased inflammatory parameters could not be observed in one patient with an RVF. Body temperature did not prove useful for early discrimination between uncomplicated cases and those with early septic complications.

**Conclusion:**

Relevant elevations of serum CRP and WBC levels were demonstrated in patients with early septic complications following surgery for colorectal DE starting at postoperative day 2–4. The cut-off value of 10 mg/dl for CRP levels may serve as an early predictor for lower GIT-related septic complications but should be used with caution in women with suspected RVF development.

**Supplementary Information:**

The online version of this article (10.1007/s00508-021-01916-w) contains supplementary material, which is available to authorized users.

## Introduction

Endometriosis represents a common disease affecting approximately 1.5% of women of reproductive age [1]. It may involve the ovaries or be detected as mere superficial peritoneal lesions; in severe cases, it may be found at the level of the uterosacral ligaments or rectovaginal septum or affect different organs, such as the urinary bladder, bowel or vagina as deep endometriosis (DE) [2–5]. Symptoms associated with colorectal DE may require surgical excision of endometriotic lesions by rectal shaving, discoid resection or segmental bowel resection. As this surgery may entail a number of complications and risk of disease recurrence, there is still an ongoing debate regarding indications for and the choice of the optimal technique [6–12]. The most frequent major complication following colonic resection for DE is anastomotic leakage (AL), which may cause peritonitis and lead to the necessity of reinterventions. Early detection is of pivotal importance since adequate treatment may minimize inflammatory reactions, thus facilitating repair and healing processes [13, 14]; however, associated clinical signs such as fever, signs of peritonitis or prolonged postoperative bowel paralysis are unspecific and may occur late accompanied by rapid clinical deterioration [15]. The C‑reactive protein (CRP) levels were found to represent an early indicator of different major surgical complications and postoperative sepsis in abdominal and colorectal cancer surgery [16–20]. CRP and white blood cell count (WBC) levels were also significantly increased in patients with major surgical complications after bowel resection for endometriosis [21]. Furthermore, a decrease of CRP from day 1 to day 3 indicated uncomplicated postoperative courses [21]. A CRP cut-off level of 100 mg/l on the 4th day was shown to predict early septic complications, such as bowel fistula, pelvic abscess and infected hematoma [22].

The aim of this study was to compare the postoperative daily changes of CRP, WBC and body temperature after bowel surgery for DE in uncomplicated cases versus cases with septic complications.

## Material and methods

This study was a descriptive, retrospective study analyzing the data of patients who consecutively underwent surgical treatment of colorectal DE between April 2015 and September 2020 at a tertiary referral center for endometriosis. Daily postoperative measurements of CRP, WBC and body temperature were performed routinely until hospital discharge. Exclusion criteria were age under 18 years, a positive history of gynecological malignancy as well as a current diagnosis or suspicion of malignancy and known chronic infectious or inflammatory diseases. Only cases with complete data on CRP and WBC levels at least for the first 3 postoperative days were included in the analyses. The study was approved by the local institutional review board (IRB). All surgical procedures were carried out by the same expert gynecological surgeon in a multidisciplinary team setting. Colorectal endometriosis was confirmed histologically in all cases included in this study. Concerning the surgical technique used for treatment of DE bowel lesions, women either underwent rectal shaving, discoid resection or segmental resection. The following information was retrieved from the patient’s charts: woman’s age, gravidity, parity, body mass index (BMI), presence of different preoperative pain symptoms such as dysmenorrhea, dyspareunia, dyschezia or dysuria, revised American Society for Reproductive Medicine (rASRM) stage, Enzian classification, CRP levels, WBC levels and body temperature as well as data on the occurrence of minor and major surgical complications as described by the Clavien-Dindo system [23].

### Statistical analysis

Data were analyzed by descriptive statistics. Patient characteristics are shown as means ± standard deviations (SD) or as relative values and percentages as appropriate. The courses of CRP, WBC and body temperature in cases without surgical complications are represented as medians together with the 25%/75% percentiles for each postoperative day, whereas the cases with surgical complications are depicted as individual curves over the hospital stay. Furthermore, sensitivity, specificity and positive and negative predictive values were calculated and a receiver operating characteristic (ROC) curve was built to verify the use of 10 mg/dl CRP as a cut-off value for detection of early septic surgical complications. IBM SPSS version 21.0 (IBM Corp., Armonk, NY, USA) and Excel from Microsoft Office version 2019 were used for data analysis.

## Results

Between April 2015 and September 2020, 183 women who did not fall under the exclusion criteria underwent surgical treatment for DE of the bowel at our institution. Of these women 10 had a major postoperative surgical complication (10/183, 5.5%). In total, four anastomotic leakages (4/183, 2.2%), two rectovaginal fistulae (RVF, 2/183, 1.1%), one abscess of the abdominal wall (1/183, 0.5%), one abscess of the vaginal cuff (1/183, 0.5%), one postoperative bleeding at the level of the abdominal wall (1/183, 0.5%) and one case of postoperative fever of unknown origin (1/183, 0.5%) were observed. Of those cases without any surgical complication, 15 cases were excluded from the analyses because of missing data on CRP and/or WBC values. Therefore, a total of 168 cases were included into the final analyses. Basic characteristics of these women are shown in Table 1. Table 2 gives an overview of the surgical procedures used for resection of colorectal DE and the respective frequencies of different complications.

**Table 1:** Characteristics of patients undergoing surgery for deep endometriosis (DE) of the bowel (*n* = 168)

Patient characteristic	Value
**Age** (years), mean ± SD	33.8 ± 5.6
**Body mass index** (kg/m^2^), mean ± SD	23.7 ± 3.9
**Gravidity**, *n* (%)	
0	129/168 (76.8)
1	32/168 (19.1)
2 or 3	7/168 (4.2)
**Parity**, *n* (%)	
Nulliparity	139/168 (82.7)
Primiparity	23/168 (13.7)
Parity of 2 or 3	7/168 (4.2)
**Preoperative pain symptom**, *n* (%)	
Dysmenorrhea	168/168 (100)
Dyspareunia	110/168 (65.5)
Dyschezia	40/168 (23.8)
Dysuria	9/168 (5.4)
**Affected Enzian compartments**, *n* (%)	
Enzian A (vagina, RVS)	134/168 (79.8)
Enzian B (USL, parametria)	145/168 (86.3)
Enzian C (rectum, sigmoid)	168/168 (100)
C1 (< 1 cm)	11/168 (6.6)
C2 (1–3 cm)	40/168 (23.8)
C3 (> 3 cm)	117/168 (69.6)
Enzian FA (adenomyosis)	86/168 (51.2)
Enzian FB (urinary bladder)	15/168 (8.9)
Enzian FU (ureters)	13/168 (7.7)
**rASRM score**, *n* (%)	
Stage 1	8/168 (4.8)
Stage 2	35/168 (20.8)
Stage 3	26/168 (15.5)
Stage 4	97/168 (57.7)

*SD *standard deviation; *RVS* rectovaginal septum; *USL* uterosacral ligaments; *rASRM score* revised American Society for Reproductive Medicine score

**Table 2:** Surgical procedures used for the resection of deep endometriosis (DE) of the bowel (*n* = 168) and the respective frequencies of different complications

	No complication (*n* = 158)	AL (*n* = 4)	RVF (*n* = 2)	Abdominal wall abscess (*n* = 1)	Other complication (*n* = 3)
**Surgical procedure**					
Shaving	8	0	0	0	1
Disc resection	23	1	0	0	1
Segmental resection	128^a^	3	2	1	1
**Protective stoma**	17	0	2	1	0

*AL* anastomotic leakage; *RVF* rectovaginal fistula

^a^One patient had both shaving and segmental resection and 2 patients had both disc resection and segmental resection

**Fig. 1:**
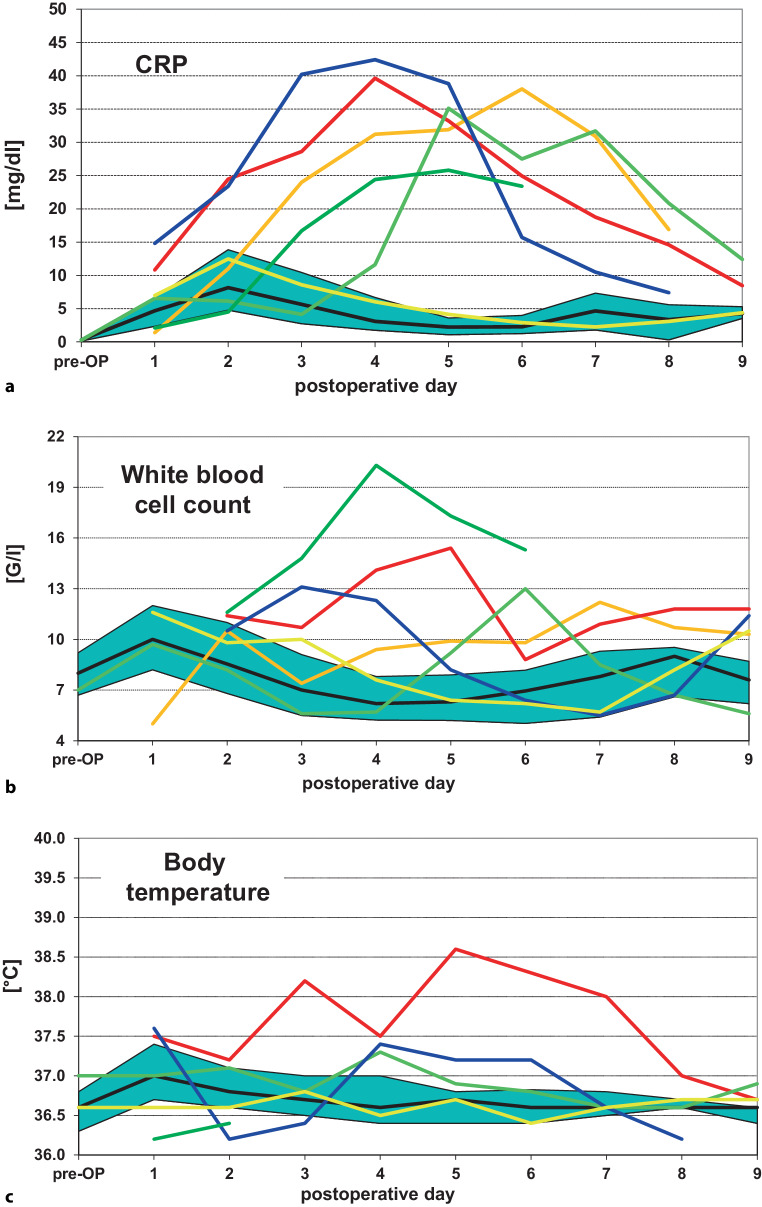
Courses of C‑reactive protein (CRP) levels (**a**), white blood cell (WBC) levels (**b**) and body temperature (**c**) over the hospital stay from preoperative values (pre-OP) to those measured on each postoperative day. The respective levels for the cases without postoperative surgical complications are shown as medians together with the 25%/75% percentiles, whereas the values for those with either an anastomotic leakage (*blue, orange, dark green and light green curves*) or a rectovaginal fistula (*red and yellow curves*) are depicted individually for each affected woman. Each color represents the same patient over **a**–**c**

Fig. 1a depicts the courses of CRP levels for all cases without any postoperative surgical complication as medians together with the 25%/75% percentiles for each day as well as for those cases with either an AL or an RVF, which are depicted as individual curves for each woman separately. Data are shown in an analogous way for WBC levels (Fig. 1b) and for body temperature (Fig. 1c). In the presence of a lower gastrointestinal tract (GIT)-related septic complication, serum CRP levels were increased over the range of the cases without surgical complications starting at postoperative days 2 to 3 (Fig. 1a). The WBC levels were increased over the normal range starting from the 3rd to 4th postoperative day and in 1 of the 4 cases with AL, they started to increase at postoperative day 5 with a peak at day 6 (Fig. 1b). One woman with RVF (yellow curve) did not show any elevation of CRP levels, WBC levels or body temperature (Fig. 1a–c). All of the cases with a surgical complication in Fig. 1a except for one case with RVF (yellow curve) showed serum CRP levels above the previously described [22] cut-off value of 10 mg/dl on the 4th postoperative day.

**Fig. 2:**
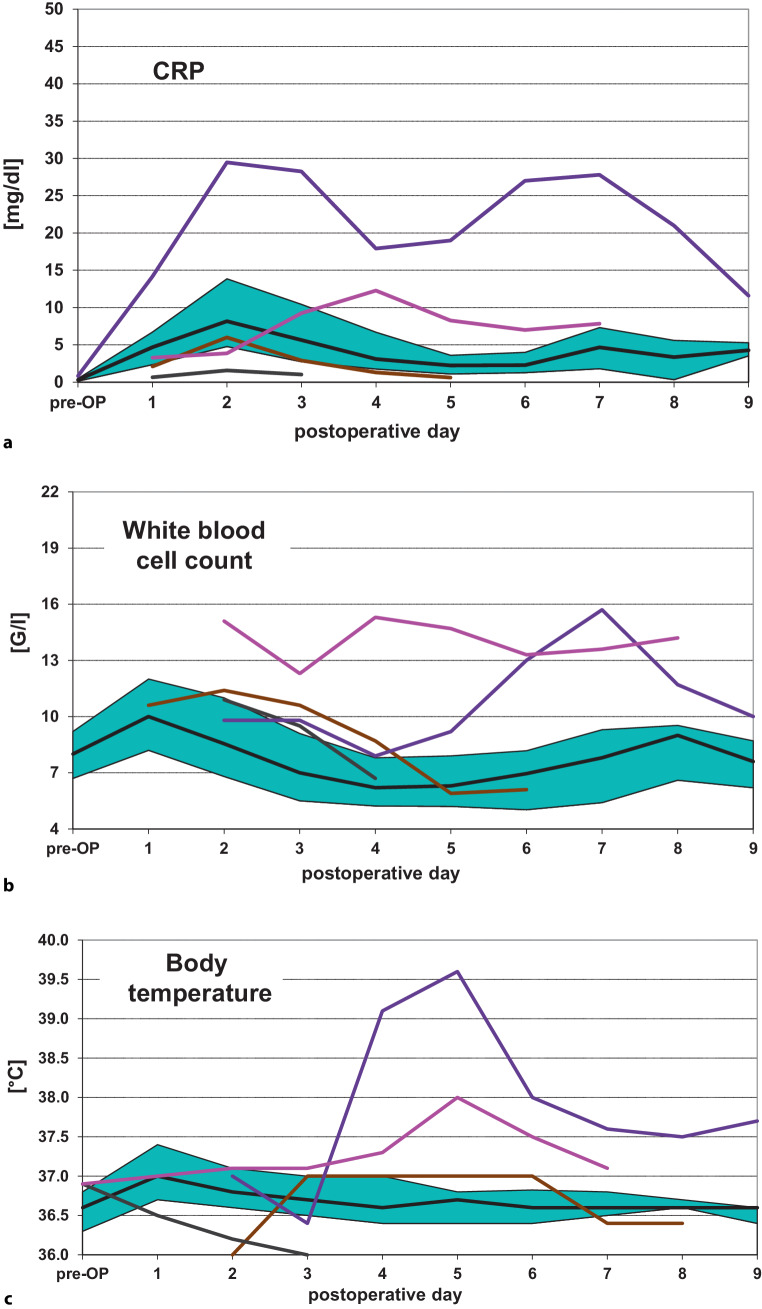
Courses of C‑reactive protein (CRP) levels (**a**), white blood cell (WBC) levels (**b**) and body temperature (**c**) over the hospital stay from preoperative values (pre-OP) to those measured on each postoperative day. The respective levels for the cases without postoperative surgical complications are shown as medians together with the 25%/75% percentiles, whereas the values for those with any postoperative surgical complication other than anastomotic leakage or rectovaginal fistula are depicted individually for each affected woman. Each color represents the same patient over **a**–**c** (*violet curve*: abscess of the abdominal wall; *purple curve*: postoperative fever of unknown origin; *brown curve*: postoperative bleeding at the level of the abdominal wall; *grey curve*: vaginal vault infection)

In Fig. 2a, the individual curves of the courses of CRP levels for each woman with postoperative surgical complications other than AL or RVF are shown. All cases without any postoperative surgical complication are again shown as medians together with the 25%/75% percentiles for each day. Data are shown in an analogous way for WBC levels (Fig. 2b) and for body temperature (Fig. 2c). The violet curve representing the data of the woman affected by a postoperative abscess of the abdominal wall is similar to those of the patients with a lower GIT-related septic complication, showing increased CRP levels starting at postoperative day 2, which are clearly higher than the cut-off level of 10 mg/dl. The patient with fever of unknown origin (purple curve) also showed CRP levels above 10 mg/dl on the 4th postoperative day, although CRP levels did not reach those observed in the other cases of septic complications.

Furthermore, a ROC curve was built for CRP levels on the 4th postoperative day (Supplemental Fig. 1). The area under the curve was 0.94. Using a cut-off value of 10 mg/dl for CRP levels for the early detection of septic complications, a sensitivity of 87.5%, a specificity of 89.9%, a PPV of 31.8% and a NPV of 99.3% were found in the present study. Excluding the only case with an RVF that showed a CRP value of only 6.02 mg/dl on postoperative day 4, a sensitivity of 100% would have been obtained for the present data, as all of the other cases of septic complications had CRP levels above 10 mg/dl on the 4th postoperative day (ranging from 11.6 to 42.2 mg/dl).

## Discussion

The present work demonstrates that serum CRP levels significantly increase in patients with major septic surgical complications following colorectal surgery for DE, starting at postoperative days 2 to 3. In contrast to previous studies [21], we observed this increase to appear later, i.e., on day 2 and 3 instead of day 2. The same was observed for serum WBC levels, which were increased over the normal range starting from the 3rd to 4th postoperative day instead of the 2nd or 3rd day [21]. In 1 of the 4 cases with AL, WBC levels started to increase even only at postoperative day 5 with a peak at day 6. Interestingly, one woman with an RVF did not show any elevation of CRP levels, WBC levels or body temperature, which is in contrast to the data published so far. The previously described cut-off level for serum CRP of 10 mg/dl on the 4th postoperative day for the detection of early septic complications [22] also fits the present data, with all of the cases with AL, 1 of the 2 cases with RVF, as well as the case of abdominal wall abscess showing CRP levels between 11.6 mg/dl and 42.4 mg/dl on postoperative day 4. Only the mentioned case with RVF would erroneously have been classified as an uncomplicated case using this cut-off value, as it showed a CRP value of 6.02 mg/dl on postoperative day 4. As a possible explanation, it could be hypothesized that the protective ileostomy also applied during the surgical procedure may have prevented clinical signs of septic response; however, the second case of RVF did exhibit significant deviations of CRP and WBC levels despite a concomitant diversion stoma which was also applied due to low anastomotic suture lines.

According to previous findings, a decrease of CRP levels from postoperative days 1 to 3 could be considered as an indicator of an uncomplicated postoperative course and therefore be used as a parameter for the decision whether early discharge from hospital after bowel resection for DE is possible [21]. In the present study, one woman with AL and another one with RVF showed a decrease in CRP levels from days 1 to 3 after surgery and would have been discharged too early if this parameter had been used for decision making, whereas all the others showed an increase in CRP levels from days 1 to 3. Interestingly, as many as 57% of all uncomplicated cases in this study showed an increase in CRP levels from days 1 to 3.

In line with the literature, body temperature did not appear to be useful for early discrimination between uncomplicated cases and those with early septic complications.

The main limitations of this study are its retrospective design and the small sample size for the complication group. Regarding its retrospective design, 15 cases had to be excluded due to missing data on serum CRP and WBC levels; however, all data for the cases with major postoperative complications were retrievable, thereby allowing a comparison with the high number of uncomplicated cases. Prospective studies have to further verify the present findings.

## Conclusion

The present work confirms the presence of relevant elevations of serum CRP and WBC levels in cases with lower GIT-related septic complications or abdominal wall abscess starting between postoperative days 2 and 4. The present data support the cut-off value of 10 mg/dl for serum CRP levels on the 4th postoperative day for the exclusion of early septic complications; however, women developing RVF may lack significant elevations of WBC and CRP. In addition, the initial decrease of inflammatory parameter levels must be interpreted with caution.

## Supplementary Information

Supplemental Fig. 1 Receiver operating characteristic (ROC) curve for the cut-off value of 10 mg/dl for serum CRP levels for detection of septic complications at postoperative day 4.
