# Supplementary material for: Serial assessment of inflammatory parameters for prediction of septic complications following surgery for colorectal endometriosis: A descriptive, retrospective study
Source: Wien Klin Wochenschr. 2021 Aug 2;134(3-4):118–24. doi: 10.1007/s00508-021-01916-w (PMC8857128; doi:10.1007/s00508-021-01916-w)
Supplement: Supplementary file 1 [file 508_2021_1916_MOESM1_ESM.odt]

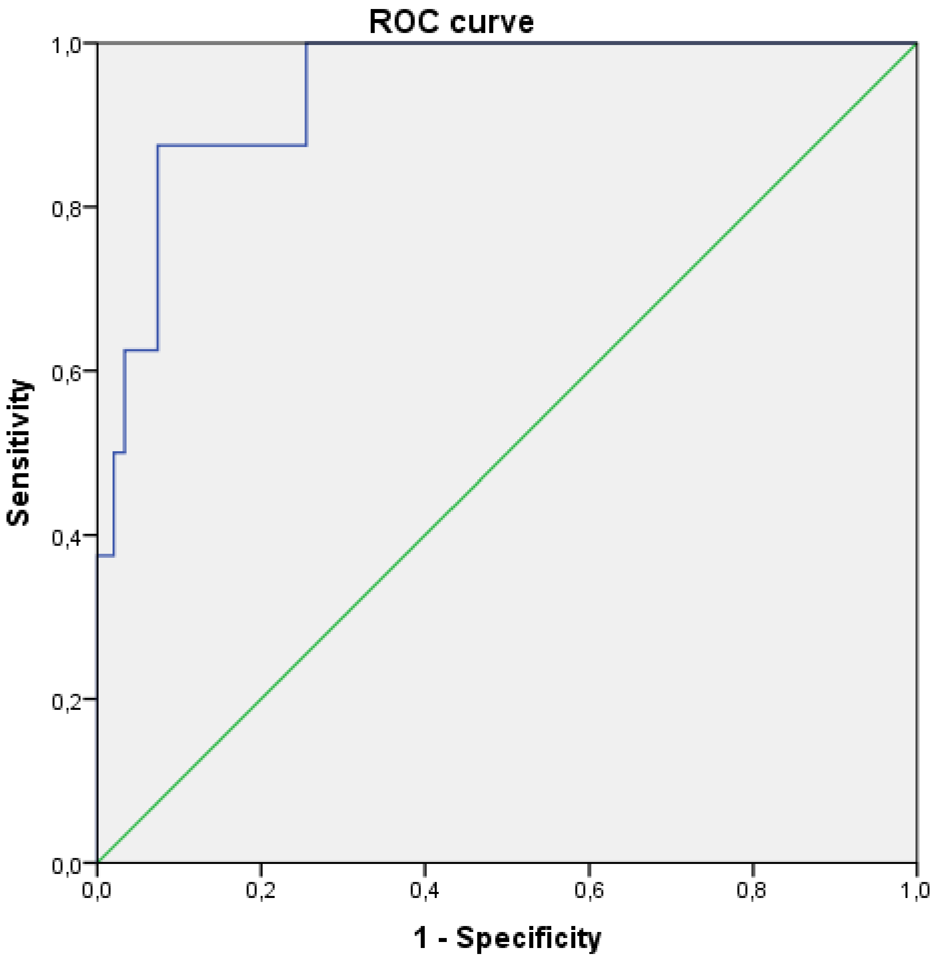
ROC curve
Sensitivity
1 - Specificity
1,0
0,8
0,6
0,4
0,2
0,0
0,0
0,2
0,4
0,6
0,8
1,0
